# Free-choice high-fat diet consumption reduces lateral hypothalamic GABAergic activity, without disturbing neural response to sucrose drinking in mice

**DOI:** 10.3389/fnins.2023.1219569

**Published:** 2023-08-03

**Authors:** Margo Slomp, Laura L. Koekkoek, Michael Mutersbaugh, Ian Linville, Serge H. Luquet, Susanne E. la Fleur

**Affiliations:** ^1^Endocrinology Laboratory, Department of Laboratory Medicine, Amsterdam UMC, Location University of Amsterdam, Amsterdam, Netherlands; ^2^Amsterdam Neuroscience, Cellular and Molecular Mechanisms, Amsterdam, Netherlands; ^3^Amsterdam Gastroenterology Endocrinology and Metabolism, Amsterdam, Netherlands; ^4^Metabolism and Reward Group, Royal Netherlands Academy of Arts and Sciences, Netherlands Institute of Neuroscience, Amsterdam, Netherlands; ^5^Department of Biomedical Engineering, University of Virginia, Charlottesville, VA, United States; ^6^Université Paris Cité, CNRS, Unité de Biologie Fonctionnelle et Adaptative, Paris, France

**Keywords:** GABA, lateral hypothalamus, free-choice high-fat diet, two-photon microscopy, sucrose drinking

## Abstract

Nutrition can influence the brain and affect its regulation of food intake, especially that of high-palatable foods. We hypothesize that fat and sugar have interacting effects on the brain, and the lateral hypothalamus (LH) is a prime candidate to be involved in this interaction. The LH is a heterogeneous area, crucial for regulating consummatory behaviors, and integrating homeostatic and hedonic needs. GABAergic LH neurons stimulate feeding when activated, and are responsive to consummatory behavior while encoding sucrose palatability. Previously, we have shown that glutamatergic LH neurons reduce their activity in response to sugar drinking and that this response is disturbed by a free-choice high-fat diet (fcHFD). Whether GABAergic LH neurons, and their response to sugar, is affected by a fcHFD is yet unknown. Using head-fixed two-photon microscopy, we analyzed activity changes in LH^Vgat^ neuronal activity in chow or fcHFD-fed mice in response to water or sucrose drinking. A fcHFD decreased overall LH^Vgat^ neuronal activity, without disrupting the sucrose-induced increase. When focusing on the response per unique neuron, a vast majority of neurons respond inconsistently over time. Thus, a fcHFD dampens overall LH GABAergic activity, while it does not disturb the response to sucrose. The inconsistent responding over time suggests that it is not one specific subpopulation of LH GABAergic neurons that is driving these behaviors, but rather a result of the integrative properties of a complex neural network. Further research should focus on determining how this dampening of LH GABAergic activity contributes to hyperphagia and the development of obesity.

## 1. Introduction

Overconsumption of highly palatable, high fat-sugary foods poses a major health risk, contributing to the development of obesity. While it is known that the brain is an important regulator of food intake and body weight, the mechanisms and circuits behind overconsumption are still elusive. Nutrition is known to influence the brain, which could subsequently alter feeding behavior. Previous research found that dietary fat intake can change neurotransmission or receptor properties in homeostatic or hedonic regions (Berland et al., 2020; Adermark et al., 2021), while sucrose consumption can lead to impairments in memory or goal-directed behaviors functions (Avena et al., 2008; Beilharz et al., 2016; Sharpe et al., 2016; Buyukata et al., 2018). Fat and sugar can also have interacting effects, where the intake of either can potentiate reward by an additive effect (DiFeliceantonio et al., 2018; Small and DiFeliceantonio, 2019; De Araujo et al., 2020; Perszyk et al., 2021). In our laboratory, we commonly use a free-choice high-fat high-sugar (fcHFHS) diet to study the additive and interacting effects of fat and sugar consumption on the brain in mice and rats (La Fleur et al., 2007, 2010; Slomp et al., 2019). This fcHFHS diet provides us the opportunity to test our hypothesis that fat and sugar have interacting effects in the brain, and together drive hyperphagia not observed when fat and sugar are consumed separately (Slomp et al., 2019).

The LH is a key area in the integration of homeostatic and hedonic information (Morales, 2022; Petzold et al., 2023), making it a prime candidate to be involved in this fat and sugar interaction effect. Neurons in the LH respond to sucrose drinking (Rossi et al., 2019; Garcia et al., 2021; Koekkoek et al., 2021), either through glucose-responsive neurons in the LH (Burdakov et al., 2005) or through taste signaling in the oral taste buds (Berk and Finkelstein, 1981; Contreras et al., 1982). High fat diet (HFD) feeding can alter glutamatergic signaling (Rossi et al., 2019; Adermark et al., 2021; Koekkoek et al., 2021) or synaptic transmission (Linehan et al., 2020) within the LH, possibly through direct effects of fatty acids (Oomura et al., 1975). The LH is a very heterogeneous collection of neurons, whose subpopulations are likely to differ in connectivity and function. Subpopulations are expressing a vast array of neuropeptides and neurotransmitters, including neurons defined by the classical neurotransmitters glutamate and γ-aminobutyric acid (GABA)(Mickelsen et al., 2019). The population of glutamatergic LH neurons (LH^Vglut^) is reported to encode satiety, and their activity is lowered by HFD feeding (Rossi et al., 2019). Previously, we observed that LH^Vglut^ neurons are responsive to sucrose drinking, and that consumption of a free-choice high-fat diet (fcHFD) disturbs this sucrose response, independent of body weight gain (Koekkoek et al., 2021). The other classical neurotransmitter population, GABAergic LH neurons, can induce consummatory behavior when stimulated, to both non-caloric and caloric stimuli (Navarro et al., 2016). Indeed, LH^Vgat^ neurons are activated during consummatory behavior, signaling palatability with increased firing rates upon sucrose consumption compared to water (Jennings et al., 2015; Garcia et al., 2021). Whether a fcHFD affects LH GABAergic activity is yet unknown, as is if fcHFD feeding changes the neural response to sucrose consumption.

Thus, a role of GABAergic LH neurons in consummatory behavior has been established, with increased neuronal responses to more palatable solutions. However, little is known about the effects of a fcHFD on the neuronal responses upon drinking sucrose. In this study, we therefore aimed to explore whether fcHFD differentially affects GABAergic LH activity in response to sucrose and water drinking. To achieve this, we used *in vivo* two-photon microscopy in Vgat^Cre^ mice, expressing Cre recombinase in neurons encoding and releasing vesicular GABA transporter (Vgat), in animals either on a regular (chow) diet or on a fcHFD.

## 2. Methods

### 2.1. Animals

Experiments were part of a student exchange program between the Amsterdam UMC and the National Institute on Drug Abuse and ethical approval was provided by the National Institute on Drug Abuse, and performed in accordance with the US National Institutes of Health Guidelines for the Care and Use of Laboratory Animals. Adult (2–4 month old) male and female heterozygous Vgat^IREScre^ (RRID:IMSR_JAX:016962; C57BL/6 J background; Stock 16,962, The Jackson Laboratory, ME, USA) mice were used. Prior to stereotaxic surgery, mice were group housed with littermates in temperature- and humidity-controlled rooms with *ad libitum* access to water and rodent chow (PicoLab Rodent Diet 20, 5,053 tablet, LabDiet/Land O’Lakes Inc., MO, USA) on a 12 h light/dark cycle.

### 2.2. Stereotaxic viral injection and GRIN lens implantation

For details on the surgical procedure, please refer to Koekkoek et al. (2021). In brief, isoflurane anesthetized mice underwent stereotaxic surgery where they first received an injection with an adeno-associated virus [rAAV2.9/CAG.FLEX-GCaMP6s.WPRE.SV40, titer: 1.34 × 1,013 genomic copies/ml; RRID:Addgene_100842; University of Pennsylvania Gene Therapy Program Vector Core, PA, USA; (18)] into the lateral hypothalamus (injection: bregma, −1.55 mm; midline, +0.90 mm; dorsal surface, −5.30 mm). After this injection, a Gradient Index (GRIN) lens (ILW-050-P146-055-NC; Go!Foton Corporation, NJ, USA) was implanted right above the viral injection site and secured to a head-bar using dental cement. After surgery, mice were individually housed for 8 weeks for post-surgical recovery, inflammatory response reduction, and to allow viral transduction.

### 2.3. Experimental paradigm

After the recovery period, the animals were individually housed in reversed light dark conditions (lights off 8 AM, on at 8 PM), and all imaging was done in the dark period. First, animals were handled daily during a week of acclimatization, after which they were assigned to either one of two diets with *ad libitum* access. The chow diet group (*n* = 4 total, 2M/2F) received chow pellets (PicoLab Rodent Diet 20, 5,053 tablet, LabDiet/Land O’Lakes Inc., MO, USA), whereas the fcHFD group (*n* = 5 total, 3M/2F) received a dish of pure beef tallow (Proper Foods For Life) in addition to their chow. Once the animals started their respective diet, water bottles were removed from their home cage to ensure motivation for drinking in the head-fixed set-up. To prevent weight loss, the chow diet group received moist chow daily to facilitate eating and hydration (the beef tallow is hydrolyzed, and fcHFD-fed animals therefore did not require any moist chow to prevent weight loss). Starting at the introduction of the diet, mice were trained daily to get used to the imaging set-up and the protocol. During the second week of diet consumption, mice underwent daily imaging sessions.

### 2.4. Two-photon fluorescence endomicroscopy system and imaging protocol

We imaged LH^Vgat^ neurons across days in awake, head-fixed mice using two-photon fluorescence endomicroscopy. For technical details regarding the two-photon microscopy set-up, refer to Koekkoek et al. (2021). ScanImage 2017b (Vidrio Technologies LLC, VA, USA) was used to collect *in vivo* imaging recordings at 1.43 Hz for all mice. On imaging days, food items were removed from the homecage for each animal 2 h before imaging onset. During daily imaging sessions, mice were placed in the imaging rig in front of a custom made lickometer that enabled the delivery of solutions and the measurement of licking using an infrared beam at the spout. At the start of the imaging session, the mice were given a 3 min baseline period, after which the trial period of the protocol started. Each trial started with a tone, followed by a 10 s window during which the mouse could trigger one single delivery (0.0128 ml) by licking the spout, and ended with 20 s no deliveries could be triggered to ensure the imaging quality. Mice could initiate a maximum of 60 trials per day, to ensure sufficient hydration, but for all analysis, the first 10 trials were used. In total, mice underwent imaging sessions for six subsequent days; with three sessions where water was delivered and three with a 10% sucrose solution delivered; all counterbalanced.

### 2.5. Functional imaging analysis

The Calcium Imaging Analysis (CaImAn) package (Pnevmatikakis et al., 2016) and Non-Rigid Motion Correction (NoRMCorre; Pnevmatikakis and Giovannucci, 2017) package were used to extract time courses from the calcium imaging data (using MATLAB R2018A, The MathWorks Inc., MA, USA). First, motion correction was performed on the recordings with the NoRMCorre code. Next, using the CaImAn package’s pipeline, regions of interest (ROI) were determined in the recordings using a constrained nonnegative matric factorization (CNMF) approach that finds the best spatial and temporal components explaining the observed fluorescence. This algorithm deals with heavily overlapping and neuropil contaminated movies, resulting in a background-corrected fluorescence signal per ROI. To be able to track the same ROI over days, recordings were manually aligned to avoid artificially increasing the number of ROIs.

To improve the signal to noise ratio, the extracted fluorescence signal was preprocessed by smoothing using a moving mean of 20 frames. After preprocessing the data, data was time-locked to the consummatory lick per trial, and each trial was subjected to visual inspection to exclude trials without a licking bout after the delivery. A 5-frame window before the delivery was used as a baseline to calculate ΔF/F, and the response was measured in the 20 frames subsequent to the delivery. Deliveries with a negative baseline value were excluded from analysis. ROIs with any ΔF/*F* value >2.5 at any time point were considered outliers, and removed for subsequent analysis. This threshold eliminated approximately 1% of measurements. We considered an average increase in ΔF/F of more than 0.05 a significant response. Therefore, neuronal responses showing an average increase in ΔF/F of more than 0.05 compared to baseline were classified as “positive,” while an average decrease in ΔF/F more than 0.05 compared to baseline was classified as “negative.” The remainder was classified as “no response.” When an individual neuron showed the same (positive/negative) response in more than half of the trials (i.e., minimally 6 out of 10), the response was considered consistent. The percentage of consistent responding neurons were averaged across the multiple imaging sessions.

### 2.6. Statistical analysis

For statistical analysis, a mixed model was used in RStudio (RStudio; https://rstudio.com/), running on R version 3.5.3 (the R Foundation, USA), using the lme4 and lmeTest packages. As a dependent variable, average fluorescence was taken, and stimulus (water/sucrose) and diet (chow/fcHFD) were taken as independent variables. To avoid artificially increasing the number of measurements, neuron number was entered as a random effect. Sex was included as a fixed factor to adjust for male/female differences. For body weight and food intake, repeated measures ANOVA was performed in Graphpad Prism 8.3.0 (Graphpad software). For consistency and number of responses, chi-square tests were performed in Prism. All bar and line graphs were made in Prism, while heatmaps were created in MATLAB. All data are plotted as mean ± SEM.

## 3. Results

### 3.1. fcHFD-feeding does not alter daily caloric intake or body weight

**Figure 1:**
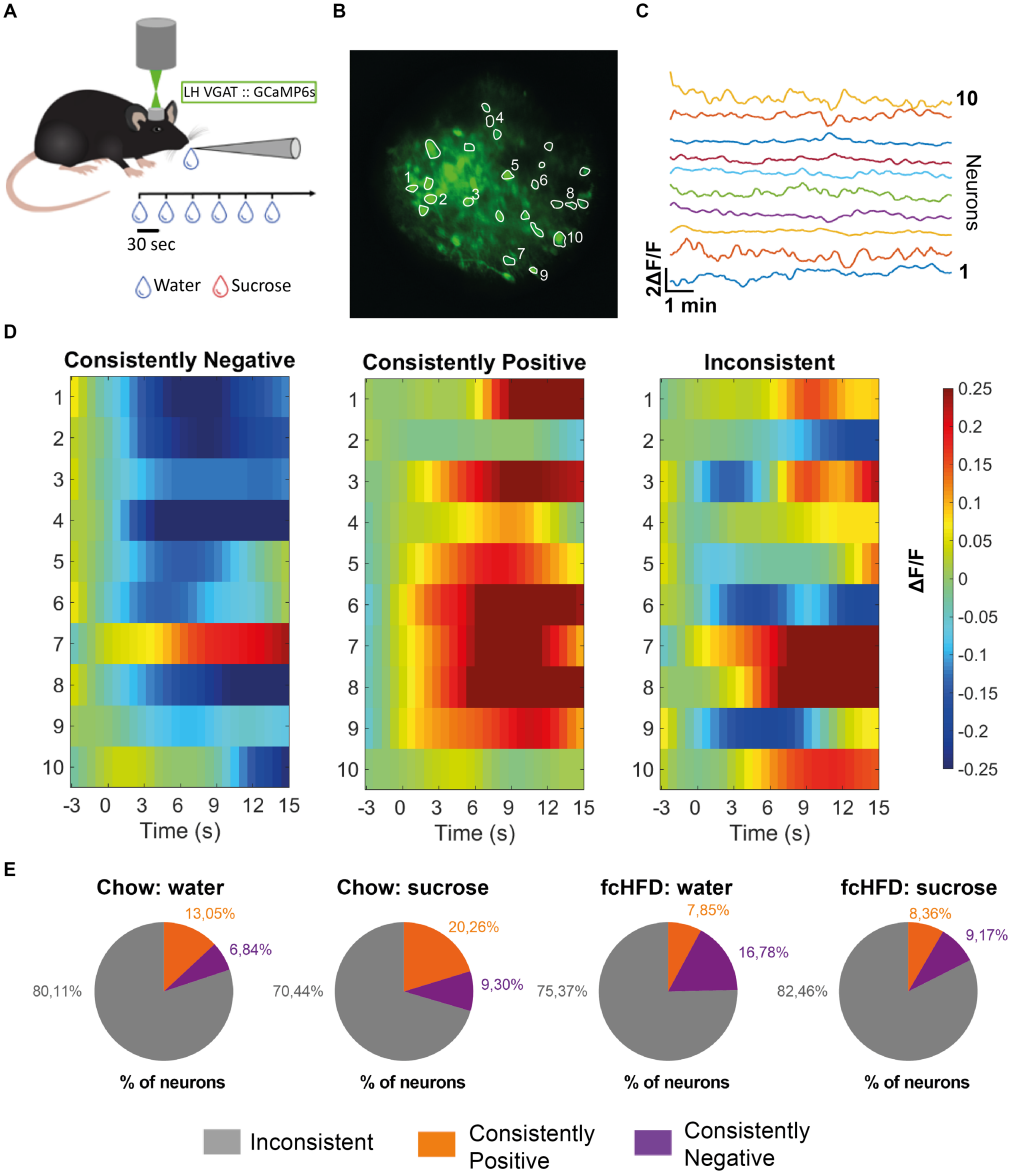
The majority of individual LH^Vgat^ neurons show inconsistent responding to water and sucrose drinking, both during chow and fcHFD feeding. **(A)** Schematic of experimental design, per imaging day there was a maximum of 60 trials, with 3 recording days per stimulus (water/sucrose). **(B)** Mean intensity map of an example field of view during imaging all included ROIs outlined, numbers indicate the 10 neurons plotted in **(C)**. **(C)** Extracted individual calcium (ΔF/F) traces per neuron showed in **(B)**. **(D)** Example heatmaps of individual neurons (over 10 trials) showing a neuron labeled as a “consistently negative” responder (left), a “consistently positive” responder (middle) and an “inconsistent” responder (right). Consistently responding was considered more than half of the trials a significant response (average ΔF/*F* > 0.05 or <−0.05). **(E)** Percentages of labeled neurons per diet group (chow/fcHFD) and consumed drink (water/sucrose). During chow feeding, the number of neurons showing a consistently positive response were increased upon sugar drinking compared to water (χ^2^ 4.29, *p*: 0.03*). During fcHFD feeding, number of consistently negative responding neurons lowered upon sugar drinking compared to water (χ^2^ 5.81, *p*: 0.02*).

Following delivery of the viral vector, adult mice expressed GCaMP6s selectively in LH^Vgat^ neurons, allowing visualization of calcium fluctuations using a GRIN lens, as a proxy for neuronal activity (Figures 1A–C). Schematic of head-fixed recording is displayed in Figure 1A, with an example mean intensity image for one FOV in Figure 1B, and accompanying example neuronal traces (preprocessed) in Figure 1C. After the experiment, viral expression and GRIN lens placement were verified using histology (Supplementary Figure S1). Mice were divided in two groups; a chow-fed (*n* = 4, 2 M/2F) and fcHFD-fed (*n* = 5, 3 M/2F) group. During 2 weeks of diet consumption, mice fed a fcHFD consumed two third of their daily calories from the fat source and decreased their chow intake resulting in a similar total caloric intake as the chow animals (Table 1). Body weight did not differ between diet groups (Table 1), however within the diet groups females had a slightly lower final bodyweight compared to males (Supplementary Table S1).

**Table 1:** Body weight at start and end of experiment, and average daily food intake of the chow and fcHFD groups.

	Chow (*n* = 4, 2 M/2F)		fcHFD (*n* = 5,3 M/2F)		Statistics
Initial weight	23.55 ± 3.99		24.18 ± 4.50		t(7) =0.02; *p* = 0.99
Final weight	21.23 ± 2.62		22.9 ± 4.17		t(7) =0.48; *p* = 0.64
Total daily calories	14.12 ± 2.51		13.96 ± 2.12		t(7) =0.02; p = 0.99
Daily calories per component	Chow	14.12 ± 2.51	Chow	4.62 ± 1.13	
			Lard	9.34 ± 1.10	

Statistics were performed by student’s *t*-test using Graphpad Prism comparing chow and fcHFD fed animals. Data displayed as mean ± SD.

### 3.2. LH^Vgat^ neurons do not respond consistently to water or sucrose over time

To determine whether there were specific neurons responding to either one of the stimuli specifically we first analyzed the responses of individual neurons over time. An individual neuronal response showing an average increase in ΔF/F of more than 0.05 compared to baseline was classified as “positive,” while an average decrease in ΔF/F more than 0.05 compared to baseline was classified as “negative.” Then, we defined a consistent response when a response from a single neuron was classified as a “positive” or “negative” response in more than half of the trials (for examples see Figure 1D). Interestingly, we found that the majority of LH^Vgat^ neurons do not consistently respond; they did not show the same response on at least 6/10 trials (Figure 1E). In the chow-fed animals, a larger percentage of LH^Vgat^ neurons responded inconsistently to water consumption (80.11%) compared to sucrose consumption (70.44%). This was accompanied by a larger number of neurons that do respond consistently positive to sucrose consumption than to water drinking (S: 20.26%, W: 13.04%, χ^2^ 4.29, *p* = 0.03*). In the fcHFD fed animals, the percentage of neurons that showed an inconsistent response was higher upon sucrose consumption (82.46%) than water (75.37%). This was driven by a significantly lower percentage of consistently negative responders during sucrose consumption than during water consumption (W: 16.78%, S: 9.17%, χ^2^ 5.81, p: 0.02*). Thus, in both diet groups and during both solutions, the majority of neurons show inconsistent responding, meaning an individual neuron does not show the same response to a stimulus (water or sucrose) over time.

### 3.3. fcHFD feeding lowers network LH^Vgat^ neuronal activity upon water or sucrose drinking

**Figure 2:**
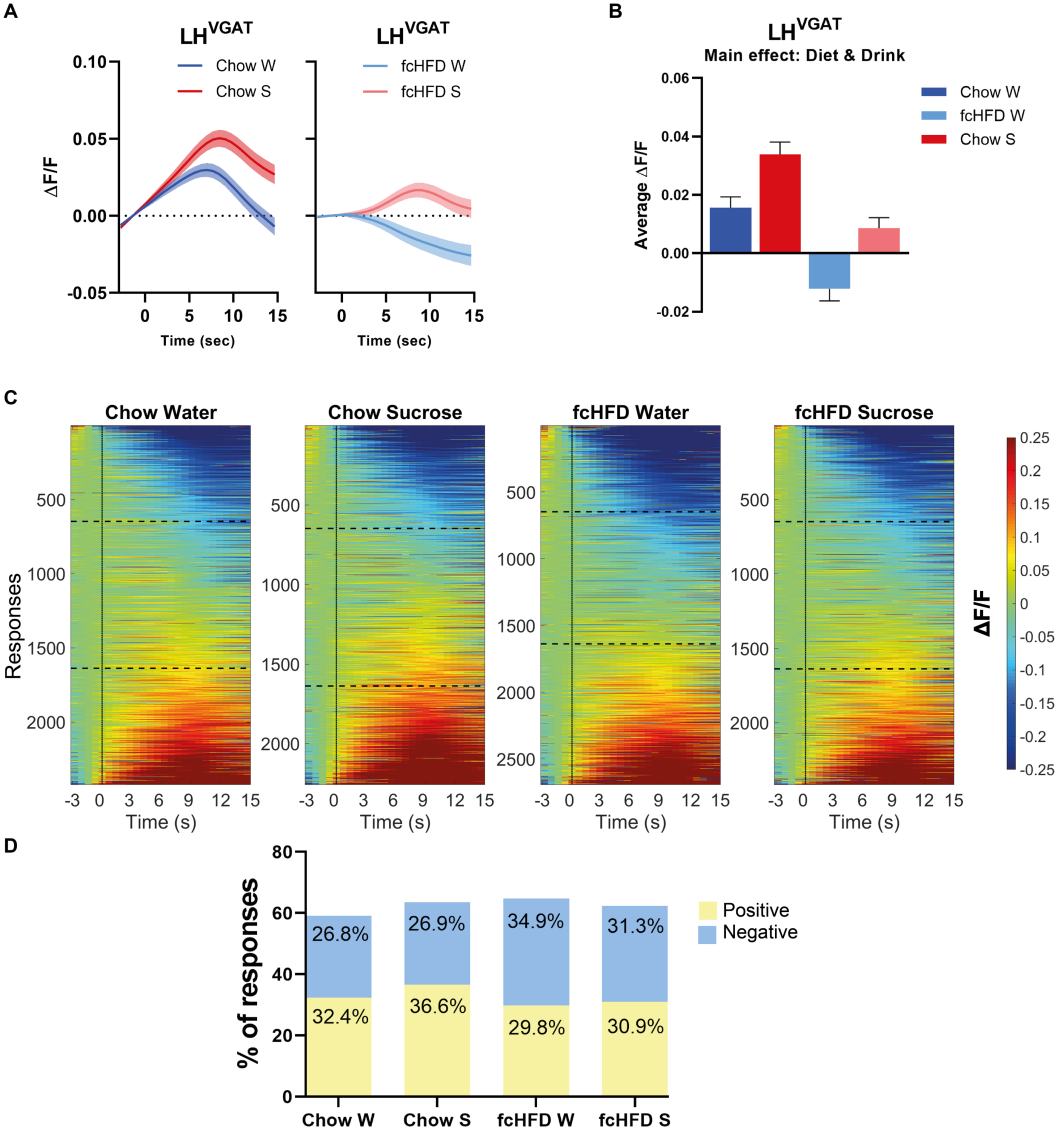
The network of LH^Vgat^ neurons responds increased to sucrose compared to water, and is dampened by fcHFD-feeding. **(A)** Average ΔF/F of all measured responses over time (i.e., 10 trials per day, 3 days per stimulus) after solution delivery at *t* = 0 for chow-fed (left) and fcHFD-fed (right) animals. **(B)** Grand average of all measurements after delivery of water (W) or sucrose (S) on both diets. **(C)** Heatmaps showing ΔF/F for all the individuals responses, with delivery at *t* = 0 (dotted line). The horizontal dotted lines represent the boundary of categorizing a response as “negative” or “positive” at an average change in ΔF/F of 0.05. **(D)** Percentage of positive, negative and no responses (as defined by threshold of ±0.05 ΔF/F) per diet and consumed drink. When chow fed, sucrose drinking significantly decreased the proportion of positive responses compared to water (χ^2^ 9.25, *p*: 0.002*), whereas during fcHFD feeding, sucrose drinking significantly decreased the proportion of negative responses (χ^2^ 7.18, *p*: 0.007*). Data displayed as mean ± SEM. *p < 0.05 as tested with a mixed effects model **(B)** or with a chi-square test **(D)**.

Since the vast majority of the individual LH^Vgat^ neurons showed inconsistent responses, all responses of all neurons, over all trials and all days, were combined to analyze the total network response. In our mixed model, we corrected for repeated measures to account for the differences in responses per neuron, and included sex as a fixed factor to account for male/female differences. When mice were fed a chow diet, the network of LH^Vgat^ neurons showed an increase to both water and sucrose consumption, but the response to sucrose was higher than to water (Figures 2A–C). fcHFD dampened the neuronal activity, but still the neurons increased their activity in response to sucrose compared to water, suggesting fcHFD does not disturb the ability to distinguish sucrose from water (Figures 2A–C). The average response from all neurons was significantly different between Diet (chow vs. fcHFD, *p* < 0.00001*) and Drink (Water vs. Sucrose, *p* < 0.0001*), as calculated by our mixed model analysis (Figure 2B). Interestingly, these differences were independent of licking behavior, as in none of the conditions licking rate was correlating with fluorescence (data not shown).

This change in average network response can be due to either a change in the number of positive or negative responses within the network, or a change in the average magnitude of the positive or negative responses. For the chow-fed animals, the increased activity upon sucrose drinking was driven by an increase in the number of neurons showing a positive response (Figure 2D, χ^2^ 9.25, *p* = 0.002). A greater number of neurons with negative responses were underlying this dampened effect of fcHFD feeding, and this proportion of negative responders was lowered during sucrose drinking (Figure 2D, χ^2^ 7.18, *p* = 0.007).

## 4. Discussion

We show here that LH^Vgat^ neurons increase their activity in response to sucrose drinking compared to water drinking, both during chow and fcHFD feeding. Interestingly, a fcHFD lowered overall activity of GABAergic LH neurons, irrespective of drink consumed. We did not find clusters of neurons specifically encoding for either one of the stimuli, as most of the neurons showed a great variety in their responses, suggesting it is more of a network response than individual neurons encoding exclusively either water or sucrose.

This study provides the first evidence that consumption of a fcHFD lowers LH^Vgat^ neuronal activity during consummatory behavior. Interestingly, this dampening effect of fcHFD feeding is independent of body weight, suggesting short-term consumption of saturated fat by itself is sufficient to alter LH GABAergic neuronal activity. HFD may have a direct effect on LH neurons, as fatty acids can increase LH neuron reactivity *ex vivo* (Oomura et al., 1975). Secondly, HFD could change the reward system by directly affecting dopamine signaling, which has been shown to be a direct target of circulating lipids (Berland et al., 2020; Wallace and Fordahl, 2022). The LH receives inhibitory input from the NAc shell, both directly from medium spiny neurons harboring dopamine receptor 1 (Drd1) and indirectly from medium spiny neurons harboring Drd2 (Castro et al., 2015; O’Connor et al., 2015; Gallo et al., 2018). The Drd2 expressing neurons within the NAc have been shown to sense triglycerides, reducing excitability of Drd2-MSNs in NAc (Berland et al., 2020). This in turn could reduce GABAergic input to the ventral pallidum (VP) which will enhance GABAergic signaling from the VP to the LH, in line with a HFD induced reduction in LH GABAergic neuronal activity. Lastly, fcHFD feeding could have altered the palatability of solutions, possibly mediated by opioid signaling. Opioid receptors within the LH regulate feeding through GABA neurons (Ardianto et al., 2016), and opioid signaling underlies taste preferences for palatable foods (Drewnowski et al., 1992). Chronic HFD consumption can decrease motivation of obtaining palatable, sweet, rewards (Arcego et al., 2020). Thus, the decrease in LH^Vgat^ neuronal activity upon fcHFD feeding during both water and sucrose drinking could be due to lowered perceived palatability, which is in line with the role of LH^Vgat^ neurons in encoding palatability (Garcia et al., 2021). However, further research is needed to find how HFD feeding is affecting GABA neurons within the LH, including specific effects in taste reactivity, palatability and dopamine and opioid signaling.

We found that LH^Vgat^ neurons increase their activity in response to sucrose, which is in line with previous research reporting a relationship between palatability (% of sucrose in solution) and LH^Vgat^ activity (Garcia et al., 2021). These authors found that LH^Vgat^ neurons signal palatability rather than satiety or hunger, which fits with the time-scale of our results, as they are unlikely to be originating from post-ingestive signaling. Data from our study were obtained from the first 10 initiated deliveries of the test-solution, and thus the response of LH neurons relies more on oral taste signals than a post-ingestive rise in extracellular glucose in the brain, which takes longer to occur (Wakabayashi and Kiyatkin, 2015). This sucrose driven increase in GABAergic LH activity could signal palatability to downstream targets. Although fcHFD reduced overall LH^Vgat^ activity fcHFD feeding does not alter the discrimination between sucrose and water, still signaling the difference in palatability between sucrose and water.

A strong advantage of two-photon imaging is the ability to determine both the response of individual neurons, as well as the entire network, over subsequent trials. We here show that only a small number of individual neurons have a specific response to water or sucrose drinking throughout multiple trials. Previous research showed that 10.1% of recorded LH^Vgat^ neurons are excited upon consumption of Ensure in a progressive ratio task (Jennings et al., 2015), and that 21% of LH^Vgat^ neurons increased their activity during sucrose drinking compared to water (Garcia et al., 2021). We report that 20.26% of neurons respond with a consistently positive response, over trials, to sucrose consumption, when chow-fed. This is comparable with the findings of Garcia et al. (2021), although we did not compare water and sucrose consumption in the same neurons, as our study design was tailored to studying the effects of fcHFD feeding. As we found that the number of consistent responding neurons was low, we propose to include both network dynamics and individual neuronal responses in future studies.

The amount of inconsistency in the response of LH^Vgat^ neurons is not surprising considering the heterogeneity of this population (Mickelsen et al., 2019). For example, NPY expressing neurons within the LH, a subpopulation of GABAergic neurons, are found to be responsive to glucose (Marston et al., 2011). Another GABAergic LH population, LepR neurons, are thought to enhance food seeking behavior, but not consummatory behavior, in a state dependent manner (Siemian et al., 2021; Petzold et al., 2023). These LepR neurons partially overlap with neurotensin (NTS) neurons, another GABAergic LH subpopulation, with a small subset being sensitive to dehydration (Brown et al., 2019). Thus, even within molecularly defined neuronal populations, there might be functional differences. The various GABAergic subpopulations can contribute to the results we observe, and imaging of subpopulations could guide to increase our knowledge about nutritional effects on the brain and how states and motivation effect LH neuronal activity upon consummatory behaviors.

In conclusion, we showed that short term fcHFD-feeding dampens overall LH GABAergic activity, while it does not disturb the ability of GABAergic cells to distinguish sucrose from water. The inconsistent responding over time, suggests that multiple subpopulations of LH GABAergic neurons are probably at play, engaging a response that results from the integrative properties of a complex neural network. To our knowledge, we are the first to describe the effects of fcHFD feeding on LH GABAergic neurons. Further research should focus on determining how this dampening of LH GABAergic activity contributes to hyperphagia and the development of obesity.

## Data availability statement

The raw data supporting the conclusions of this article will be made available by the authors, without undue reservation.

## Ethics statement

The animal study was approved by the National Institute on Drug Abuse. The study was conducted in accordance with the local legislation and institutional requirements.

## Author contributions

MS: methodology, formal analysis, investigation, software, visualization, and writing – original draft. LK: conceptualization, methodology, formal analysis, investigation, visualization, and writing – review and editing. MM and IL: software. SL: writing –review and editing. SF: conceptualization, methodology, writing – review and editing, and supervision. All authors contributed to the article and approved the submitted version.

## Funding

This work was supported by an AMC PhD fellowship grant awarded by the AMC Executive Board and by the Netherlands Organization for Health Research and Development (Aspasia grant 015.012.005).

## Conflict of interest

The authors declare that the research was conducted in the absence of any commercial or financial relationships that could be construed as a potential conflict of interest.

## Publisher’s note

All claims expressed in this article are solely those of the authors and do not necessarily represent those of their affiliated organizations, or those of the publisher, the editors and the reviewers. Any product that may be evaluated in this article, or claim that may be made by its manufacturer, is not guaranteed or endorsed by the publisher.
