# Supplementary material for: Free-choice high-fat diet consumption reduces lateral hypothalamic GABAergic activity, without disturbing neural response to sucrose drinking in mice
Source: Front Neurosci. 2023 Aug 3;17:1219569. doi: 10.3389/fnins.2023.1219569 (PMC10434857; doi:10.3389/fnins.2023.1219569)
Supplement: Supplementary file 1 [file Data_Sheet_1.docx]

Supplementary Material

Free-choice high-fat diet consumption reduces lateral hypothalamic GABAergic activity, without disturbing neural response to sucrose drinking in mice

M.Slomp^1,2,3,4†^, L.L.Koekkoek^1,2,3,4†^, M.Mutersbaugh^5‡^, I.Linville^5^, S.H. Luquet^6^, S.E. la Fleur^1,2,3,4*^

*** Correspondence:** S.E. la Fleur, [s.e.lafleur@amsterdamumc.nl](mailto:s.e.lafleur@amsterdamumc.nl)


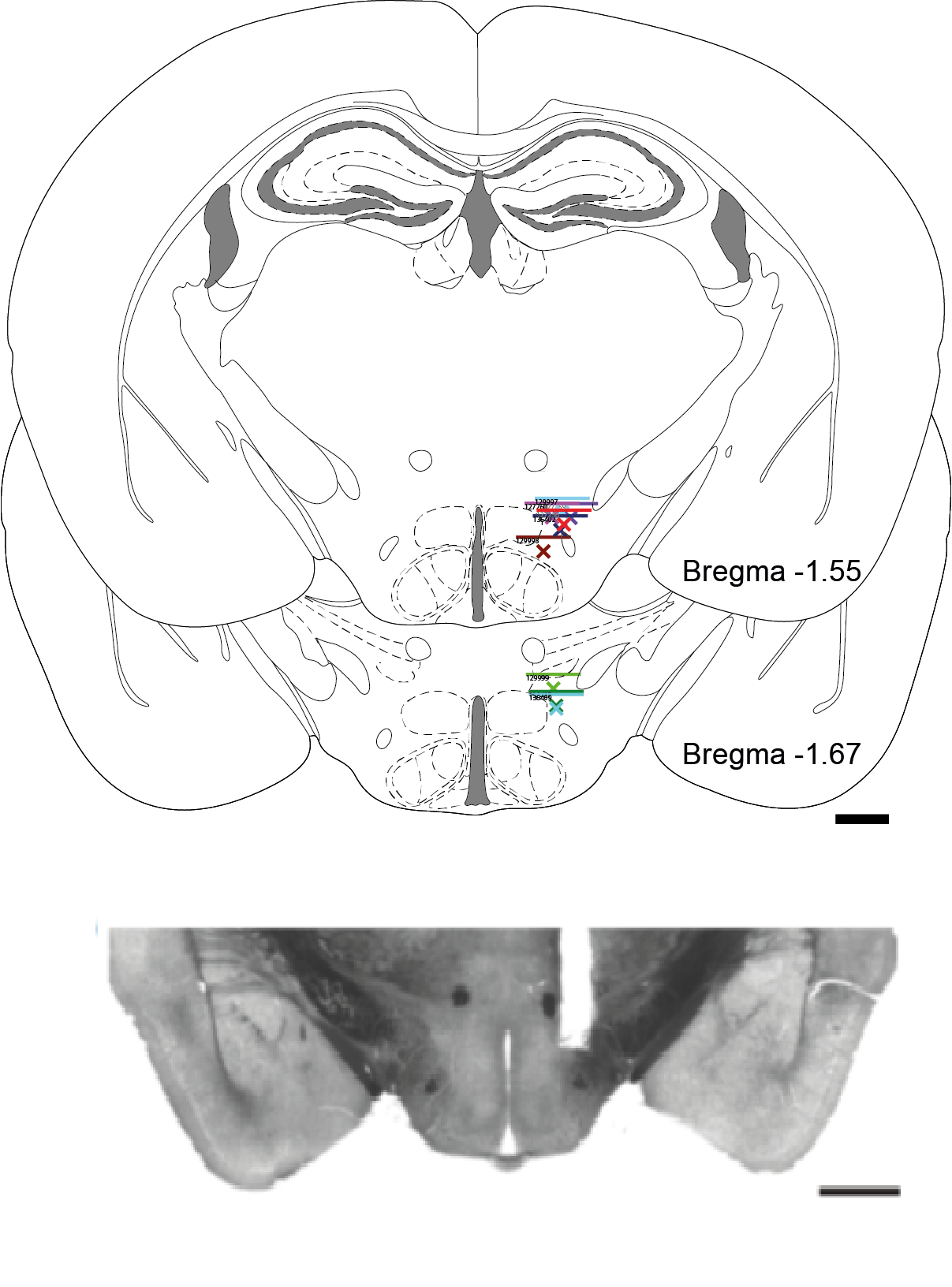


**Figure S1. GRIN lens placement of included animals.** Representative darkfield image demonstrating lens placement. Lines indicate bottom of the lens and x’s indicate working distance (approx. 130µm). Scale bar schematics = 500 µm.

**Table S1. Individual values for initial and final bodyweight, and average daily food intake of the chow and fcHFD groups.** No statistics performed due to the limited sample size.

|  |  | Females |  | Males |  |  |
| --- | --- | --- | --- | --- | --- | --- |
| Chow | |  |  |  |  |  |
| Initial weight | | 21.1 | 19.7 | 24.8 | 28.6 |  |
| Final weight | | 19.0 | 19.0 | 22.8 | 24.1 |  |
| Total daily calories | | 13.21 | 15.47 | 16.76 | 11.06 |  |
|  |  |  |  |  |  |  |
| fcHFD |  |  |  |  |  |  |
| Initial weight | | 19.2 | 20.8 | 23.2 | 30.1 | 24.2 |
| Final weight | | 18,6 | 20.0 | 21.9 | 28.6 | 22.5 |
| Total daily calories | | 12.98 | 14.88 | 14.15 | 16.83 | 11.89 |
| Daily calories per component | Chow | 4.39 | 3.41 | 5.04 | 5.87 | 3.19 |
|  | Lard | 8.59 | 11.47 | 9.11 | 10.96 | 8.70 |
